# The dietary antioxidant quercetin reduces hallmarks of bleomycin-induced lung fibrogenesis in mice

**DOI:** 10.1186/s12890-020-1142-x

**Published:** 2020-04-29

**Authors:** Agnes W. Boots, Carmen Veith, Catrin Albrecht, Roger Bartholome, Marie-José Drittij, Sandra M. H. Claessen, Aalt Bast, Martin Rosenbruch, Leonie Jonkers, Frederik-Jan van Schooten, Roel P. F. Schins

**Affiliations:** 1 Department of Pharmacology and Toxicology, NUTRIM School of Nutrition and Translational Research in Metabolism, Faculty of Health, Medicine and Life Sciences, Maastricht University, Universiteitssingel 50, 6229 ER Maastricht, The Netherlands; 2 IUF - Leibniz Research Institute for Environmental Medicine, Auf’m Hennekamp 50, 40225 Düsseldorf, DE Germany; 3 Heinrich- Heine University, Düsseldorf, Germany

**Keywords:** Bleomycin, Dietary supplementation, Inflammation, Mice, Oxidative stress, Quercetin

## Abstract

**Background:**

Idiopathic pulmonary fibrosis (IPF) is a chronic, lethal disease of which the etiology is still not fully understood. Current treatment comprises two FDA-approved drugs that can slow down yet not stop or reverse the disease. As IPF pathology is associated with an altered redox balance, adding a redox modulating component to current therapy might exert beneficial effects. Quercetin is a dietary antioxidant with strong redox modulating capacities that is suggested to exert part of its antioxidative effects via activation of the redox-sensitive transcription factor Nrf2 that regulates endogenous antioxidant levels. Therefore, the aim of the present study was to investigate if the dietary antioxidant quercetin can exert anti-fibrotic effects in a mouse model of bleomycin-induced pulmonary fibrogenesis through Nrf2-dependent restoration of redox imbalance.

**Methods:**

Homozygous Nrf2 deficient mice and their wildtype littermates were fed a control diet without or with 800 mg quercetin per kg diet from 7 days prior to a single 1 μg/2 μl per g BW bleomycin challenge until they were sacrificed 14 days afterwards. Lung tissue and plasma were collected to determine markers of fibrosis (expression of extracellular matrix genes and histopathology), inflammation (pulmonary gene expression and plasma levels of tumor necrosis factor-α (TNFα) and keratinocyte chemoattrachtant (KC)), and redox balance (pulmonary gene expression of antioxidants and malondialdehyde-dG (MDA)- DNA adducts).

**Results:**

Mice fed the enriched diet for 7 days prior to the bleomycin challenge had significantly enhanced plasma and pulmonary quercetin levels (11.08 ± 0.73 μM versus 7.05 ± 0.2 μM) combined with increased expression of Nrf2 and Nrf2-responsive genes compared to mice fed the control diet in lung tissue. Upon bleomycin treatment, quercetin-fed mice displayed reduced expression of collagen (COL1A2) and fibronectin (FN1) and a tendency of reduced inflammatory lesions (2.8 ± 0.7 versus 1.9 ± 0.8). These beneficial effects were accompanied by reduced pulmonary gene expression of TNFα and KC, but not their plasma levels, and enhanced Nrf2-induced pulmonary antioxidant defences. In Nrf2 deficient mice, no effect of the dietary antioxidant on either histology or inflammatory lesions was observed.

**Conclusion:**

Quercetin exerts anti-fibrogenic and anti-inflammatory effects on bleomycin-induced pulmonary damage in mice possibly through modulation of the redox balance by inducing Nrf2. However, quercetin could not rescue the bleomycin-induced pulmonary damage indicating that quercetin alone cannot ameliorate the progression of IPF.

## Background

IPF is a progressive and often lethal disease with a european prevalence of 23 cases and incidence of up to 7 cases per 100.000 and a median survival of 3–5 years after diagnosis [1, 2]. IPF prevalence and incidence, both positively correlated with age, have increased during the recent years, presumably due to the rapid expansion of the elderly population [1, 3]. Key clinical feature of IPF is a cruelly impaired lung function, characterized by progressive dyspnea (difficulty breathing) and a non-productive cough, resulting in a drastically reduced vital capacity and thus quality of life [4]. Current IPF treatment involves two newly FDA-approved drugs pirfenidone and nintedanib that are capable of slowing down the progression of the disease but cannot stop nor cure the disease [5–8]. Additionally, they are not equally effective in all patients and it is still not clear which patient would benefit best from what drug. Consequently, there is an urgent need for additional effective therapy options [6]. However, in order to meet this necessity, more knowledge regarding the exact pathogenesis of IPF is compulsory.

The pathogenesis of IPF is a combination of genetic predisposition and common environmental triggers, including bacteria and cigarette smoke [2, 9], leading to multiple and continuous insults to the lung epithelium. Such insults will cause early onset inflammation shading off into impaired wound healing and excessive tissue scarring. Main characteristic of such insults is a disturbed redox balance [10, 11], i.e. an overload of reactive oxygen species (ROS) in comparison to the presence of protective antioxidants. An increased oxidant burden will cause severe oxidative damage and trigger up-regulation of a number of genes involved in inflammation and/or fibrogenesis [11]. Besides being a pathological feature of IPF, as can be deducted from the higher levels of various oxidative damage markers in these patients including exhaled ethane, nitric oxide, malondialdehyde levels and elevated urinary isoprostane levels [12–15], oxidants are also an important player in the development of the disease [16]. Indeed, oxidative stress mediates several epithelial and fibroblastic alterations that influence lung homeostasis and hamper pulmonary repair and regeneration pathways. Interestingly, these oxidative changes will be further enhanced by the age-dependent increased oxidant production associated with IPF, thereby contributing to pro-fibrotic conditions [11, 16].

To offer protection against oxidant-induced damage, our body comprises an elaborate antioxidant defense system that is largely controlled by antioxidant genes [13, 17]. Upon activation by oxidants, the redox-sensitive transcription factor Nrf2 induces the transcription of antioxidants as a feedback mechanism to restore systemic redox balance and maintain homeostasis [18]. Indeed, exposure to oxidative stimuli such as cigarette smoke and particulate matter up-regulates Nrf2-dependent antioxidant genes in mice carrying the functional transcription factor [19–21]. Moreover, the loss of Nrf2 results in the development of accelerated, more severe lung emphysema, oxidative stress, epithelial damage and enhanced sensitivity towards oxidants after exposure to oxidative stimuli [18, 22, 23]. In IPF, an increased pulmonary Nrf2 expression as well as elevated antioxidant levels in the broncho-alveolar fluid are reported [24], suggesting the existence of a compensatory, Nrf2-driven feedback mechanism that does increase the pulmonary antioxidant levels but not to the extent that they can completely restore systemic redox balance and maintain homeostasis in IPF.

Due to the large involvement of ROS in the pathophysiology of IPF, some antioxidants including N-acetylcysteine (NAC) [25] and pirfenidone [5] have already been implemented as therapy in order to reduce the increased oxidative burden in IPF. However, although NAC supplementation reduced collagen deposition in bleomycin exposed rats [26], purely oxidant scavenging has not been enough to completely cure or prevent IPF until now [8]. Consequently, there is an urgent need for an additional therapeutic approach that will exert health beneficial effects by modulating the important oxidant-related pro- and antifibrotic pathways rather than by solely scavenging antioxidants. A good candidate for such an approach would be a non-pharmacological compound with a known working mechanism that compiles of more than purely scavenging capacities. The dietary antioxidant and redox modulator quercetin could qualify as such a suitable alternative.

Quercetin is a member of the flavonoids family, dietary antioxidants that are ubiquitously present in vegetables, fruit, tea and wine [17]. The daily Dutch intake has been estimated at several hundreds of mg/day [27] of which the subfamily flavones and flavonols to which quercetin belongs delivers up to 16 mg/day [27]. The most abundant flavonol is quercetin, representing 70% of this intake, which has been shown to act as a powerful phenolic antioxidant both in vitro and in vivo [17, 27]. By scavenging oxidants, quercetin can offer direct protection against both oxidative damage and inflammation, two key processes in the development of lung fibrosis. Indeed, we have already demonstrated that quercetin reduces markers of oxidative stress and inflammation in IPF and sarcoidosis [28, 29].

Additionally, quercetin may indirectly improve the antioxidant defense by increasing Nrf2-driven antioxidant production. Oxidants activate Nrf2 by oxidizing its inhibitory protein Keap1, after which Nrf2 is released and translocated to the nucleus [19, 30]. In the nucleus, Nrf2 binds to the antioxidant response element (ARE) and thus activates various antioxidant-genes including superoxide dismutase, glutathione peroxidase and heme oxygenase 1 [28, 30]. Interestingly, Nrf2 is suggested to be a specific target for phenolic antioxidants [30] presumably via its release from Keap1 upon thiol arylation [31]. In that way, quercetin will not only protect against a-specific damage caused by increased oxidant levels but will also further increase specific protection against these oxidants.

The aim of our study was to investigate if the dietary antioxidant quercetin can exert anti-fibrotic effects in a mouse model of bleomycin-induced pulmonary fibrogenesis through Nrf2-dependent restoration of redox imbalance.

## Methods

### Mouse model

Experiments were performed using homozygous Nrf2 deficient (Nrf2^−/−^) mice that were originally produced in the laboratory of M. Yamamoto [32] and backcrossed to C57BL/6 J at least 9 times. The knockout breeder mice were purchased from Riken BioResource, Tsukuba, Japan. The homozygous mutant (Nrf2^−/−^) and wildtype (Nrf2^+/+^) littermates from both genders, produced from heterozygous breeding, were used in the present study at an age of 10–12 weeks. A pilot kinetic study was performed in a limited number of animals, using C57BL/6 J mice exclusively as no effect of Nrf2 deficiency on the intestinal uptake kinetics of quercetin was anticipated. Animals were kept in an in-house pathogen-free and environmentally controlled facility (safety level 1) according to guidelines of the Society for Laboratory Animals Science (GVSOLAS). A 12 h light/dark cycle was maintained and food and water provided ad libitum. The animal study was carried out following approval by the North Rhine Westphalia State Agency for Nature, Environment and Consumer Protection (NRW-LANUV (Az.: 87–51.04.2010.A276)) under the provisions of the German Animal Protection Law (*Tierschutzgesetz: 18 Mai 2006 (BGBl.IS.1206.1313)*. The study was conducted following the principle of the 3Rs (refinement, reduction and replacement), minimizing the pain and using standardized approved procedures for analgesia and euthanasia according to the Law.

### Quercetin diet and evaluation of effectiveness

Animals had ad libitum access to either the control diet (AIN893, ssniff Spezialdiäten, Soest Germany) or the control diet enriched with 800 mg quercetin per kg diet (Merck, Darmstadt, Germany) starting from 7 days prior to the bleomycin challenge until they were sacrificed. Quercetin was stabilized (I) by adding 0.1 g citric acid per liter water while preparing both diets and (II) by replacing food pellets by a freshly thawed stock on a daily basis while providing both diets. Based on a daily intake of 5 g food per day and an average weight of 20 g, this quercetin-enriched diet will result in an average daily intake of 200 mg quercetin/kilogram BW. Weight gain, general signs of (dis) comfort and illness as well as survival were recorded on a daily basis throughout the entire dietary supplementation to ensure the safety of this intervention. To evaluate its effectiveness, six C57BL/6 J mice were fed for 1 week with quercetin enriched or control diet and sacrificed to determine plasma and pulmonary quercetin concentrations as well the expression of Nrf2 and Nrf2-responsive antioxidant genes.

### Intervention study

After 1 week of quercetin-enriched or control diet, Nrf2^−/−^ and Nrf2^+/+^mice were exposed to 1 μg/2 μl per gram BW bleomycin in 0.9% NaCl or 0.9% NaCl by a single pharyngeal aspiration under isoflurane anaesthesia. After 14 days, animals were brought under deep anaesthesia using 50 mg/kg body weight pentobarbital and euthanized via exsanguination after which blood and lungs were collected by respectively cardiac puncture and surgical removal. Left lungs were used for histology and processed as described below. The lobes of the right lungs were equally divided in 2 parts, one for RNA extraction and one for homogenate preparation, and snap-frozen until further analysis. Blood was centrifuged (10′, 1200 g, 4 °C) after which plasma aliquots were also snap-frozen until further analysis.

**Fig. 1:**
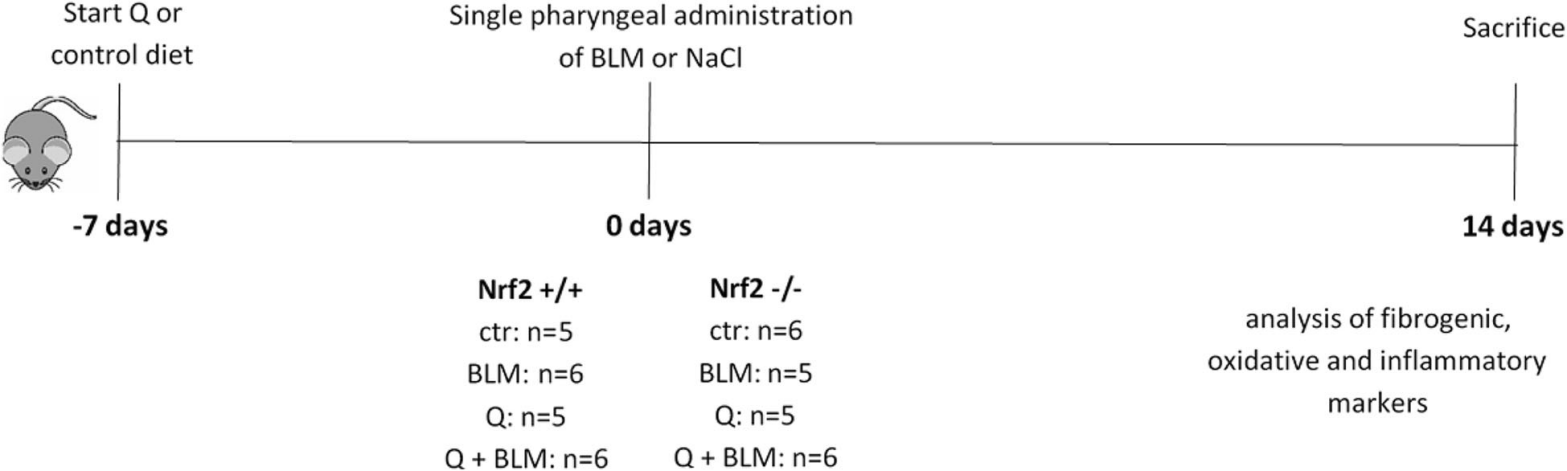
Schematic overview of the study design (Q = quercetin; BLM = bleomycin)

A schematic overview of the experimental setup, including the number of animals per group, is provided in Fig. 1. For all groups, littermates were used to ensure optimal comparison of the influence of both factors on the primary and secondary endpoints of this study.

### Histopathological evaluation of lung tissues

Left lungs were fixed in 4% paraformaldehyde /PBS, pH 7.4, dehydrated in a series of ethanol and subsequently xylol and embedded in paraffin. Sections of the left lungs were cut at a thickness of 4 μm, rehydrated and stained with H&E (hematoxylin and eosin) and Masson’s Trichrome. Subsequently, slides were evaluated histopathologically applying a semiquantitative grading: 1 = minimal, 2 = slight, 3 = marked, 4 = severe, 5 = massive.

### RNA isolation

To isolate RNA from mouse lungs, the tissue was first homogenized using a tissue homogenizer in 700 μL Qiazol (Invitrogen, Carlsbad, CA). Afterwards, the samples were incubated for 5 min at room temperature, 150 μL chloroform were added and the solutions were mixed. The samples were centrifuged for 15 min at 12.000 rpm and the upper aqueous layer was used for RNA isolation.

RNA was isolated and purified using the RNeasy mini kit (Qiagen, Venlo, the Netherlands) according to the manufacturer’s instructions. A nanodrop spectrophotometer (Thermo Scientific, Waltham, MA) was used to determine the RNA concentration.

### Real-time PCR

cDNA was synthesized from 300 ng isolated RNA using IScript (Biorad, Hercules, CA) according to the manufacturer’s instructions. Next, RT-PCR was performed using SYBR Green PCR Supermix (BioRad) with 2.5 μL of 10 times diluted cDNA and 0.4 μmol/L predesigned primers. PCR amplifications were carried at 95 °C for 10 s for denaturation and 40 cycles of annealing/elongation (60 °C, 30 s) for selected genes (Table 1). The gene expression was normalized to the house keeping gene β-actin and quantified according to the 2^−∆∆Ct^ method to relatively quantify the expression of the genes of our interest. The influence of quercetin on the redox-effects of bleomycin was analyzed by measuring the expression of Nrf2 and the Nrf2-responsive genes HO-1, ƴ-GCS, SOD2, and CAT. The alleged anti-inflammatory effects of the dietary intervention were investigated by including CXCL-1 (KC) and TNF-α, whereas COL1A2 and FN1, were added as pro-fibrotic genes of interest.

**Table 1:** Mouse RT-PCR forward and reverse primer sequences

Gene of interest	Forward primer	Reverse primer
β-actin	CTGAATGGCCCAGGTCTGA	CCCTCCCAGGGAGACCAA
NRF2	GCAGGCTATCTCCTAGTTCTCC	GCTACTTGCAGCAGAGGTGA
HO-1	GAGCCTGAATCGAGCAGAAC	CCTTCAAGGCCTCAGACAAA
y-GCS	TGCAGGAGCAGATTGACAGG	TAGAGAAAGCAAGCGGGTGG
SOD2	GGCCAAGGGAGATGTTACAA	ACCCTTAGGGCTCAGGTTTG
CAT	AGCGACCAGATGAAGCAGTG	TCCGCTCTCTGTCAAAGTGTG
CXCL1 (KC)	GGTGAGGACATGTGTGGGAG	CGAGACCAGGAGAAACAGGG
TNF-α	CAGCGCTGAGGTCAATCTGCC	TGCCCGGACTCCGCAA
COL1A2	GCAGGTTCACCTACTCTGTCCT	CTTGCCCCATTCATTTGTCT
FN1	CCCTGTTCTGCTTCAGGGTT	AAAGCAGAGGTGTCTGGGTG

### Homogenate preparation

From the collected right lung tissue, homogenates were made by crushing with liquid nitrogen. Before crushing, the weight of the tissue was determined. Afterwards, 3 parts of sodium phosphate buffer (145 mM, pH = 7.4) was added to the crushed tissue and protein content was measured using Pierce BCA protein assay kit according to manufacturer instructions.

### Determination of total plasma quercetin concentration

Total plasma quercetin concentration was measured as previously described [33] after enzymatic hydrolysis using high performance liquid chromatography (HPLC) with colorimetric array-detection.

### Luminex (Bio-Plex cytokine assay)

Plasma cytokine profiles were determined with Bio-Plex assays (Bio-Rad) using Luminex xMAP-technology. To quantify the concentrations of 11 different cytokines, we used a Bio-Plex murine cytokine multiplex panel including IL-4, TNFα, KC, MIP2, MCP-1, IL-13, IL-1β, IL-17a, IL-10, MIP1-α and MIP-1β. All assays were performed as described by the manufacturer’s instructions. Briefly, 50 μL of the antibody magnetic beads were added to each well and unbound antibody was removed. Next, 25 μL plasma were added to 25 μL universal assay buffer and incubated for 2 h. The plate was washed and 25 μL detection antibody, which binds to its corresponding analyte present in the sample, were added for 30 min. 50 μL Streptavidin-PE were added after washing and incubated for 30 min. After washing, the beads were resuspended in 120 μL reading buffer. Next, the bead and reporter quantity of the antibody sandwich formed around the analyte was determined by a laser detector. Finally, the beads and reporter quantities measured were compared to those of an internal standard corresponding to each specific cytokine. Data analysis was done with a Luminex 100 IS 2.3 system using the Bio-Plex Manager 4.1.1 software.

### Trolox antioxidant capacity

The trolox equivalent antioxidant capacity (TEAC value) is a measurement for the total antioxidant status, assessing the capacity of a compound to scavenge ABTS radicals. This assay has been performed as described earlier [34] with some minor modifications. In short, blood was centrifuged (3000 rpm, 5′ at 4 °C) and the obtained plasma deproteinized by adding 10% TCA (1:1) before centrifugation (13.000 rpm, 5′ at 4 °C). To generate ABTS radicals (ABTS^•^), a solution of 0.23 mM ABTS (2,2′-azinobis-(3-ethylbenzothiazoline-6-sulfonic acid)) and 2.3 mM ABAP (2,2′-Azobis-(2-amidinopropane)HCl) was incubated at 70 °C until the absorbance at 734 nm reached 0.70 ± 0.02. The antioxidant capacity of the samples was measured by mixing 50 μl of the plasma sample with 950 μl ABTS^•^ solution followed by 5 min incubation at 37 °C. The decrease in absorbance was measured at 734 nm and the trolox equivalent of the samples was calculated using the absorbance of trolox standards.

### Malondialdehyde-dG DNA adducts (M_1_-dG)

The presence of M_1_-dG as was evaluated using 32P-postlabeling as previously described (Peluso et al., 2013). To this end, DNA was first hydrolysed by incubating in 2.5 mM calcium chloride for 4.5 h at 37 °C (pH 6.0) in the presence of 21.45 mU/μl micrococcal nuclease and 6.0 mU/μl spleen phosphodiesterase in 5.0 mM sodium succinate. Upon hydrolysis, samples were incubated with 0.1 U/μl NP1 in 46.6 mM sodium acetate (pH 5.0) and 0.24 mM ZnCl_2_ for 30 min at 37 °C. Next step after this NP1 treatment was the generation of ^32^ P-labelled DNA adducts in bicine buffer (20 mM bicine, 10 mM MgCl_2_, 10 mM dithiotreithol, 0.5 mM spermidine, pH 9.0) by adding 1.8 μl of 0.16 mM Tris base and incubating at 37 °C for 30 min with 7–25 μCi of carrier-free [ƴ-^32^ P] ATP (3000 Ci/mM) and polynucleotide kinase T4 (0.75 U/μl). These generated ^32^ P-labeled samples were then applied to polyethyleneimine cellulose thin layer chromatography plates (Macherey-Nagel, Germany) and resolved in a low-urea solvent system as previously described [35]. Storage phosphor imaging techniques employing intensifying screens from Molecular Dynamics (Sunnyvale, California) were used to detect and quantify M_1_–dG adducts as well as normal nucleotides, i.e. diluted untreated samples. Levels of M_1_-dG adducts were expressed as relative adduct labelling, i.e. as pixels in adducted nucleotides/pixels in normal nucleotides, and corrected across experiments based on the recovery of an MDA-treated DNA adduct standard.

### Statistics

All quantitative data are represented as means ± SEM. Statistical differences between groups were evaluated by the nonparametric Mann–Whitney U-test using GraphPad Prism software (version 7.3; GraphPad Software, La Jolla, CA), and considered significant at a *P* value of less than 0.05.

## Results

### Effectiveness of quercetin supplementation

**Fig. 2:**
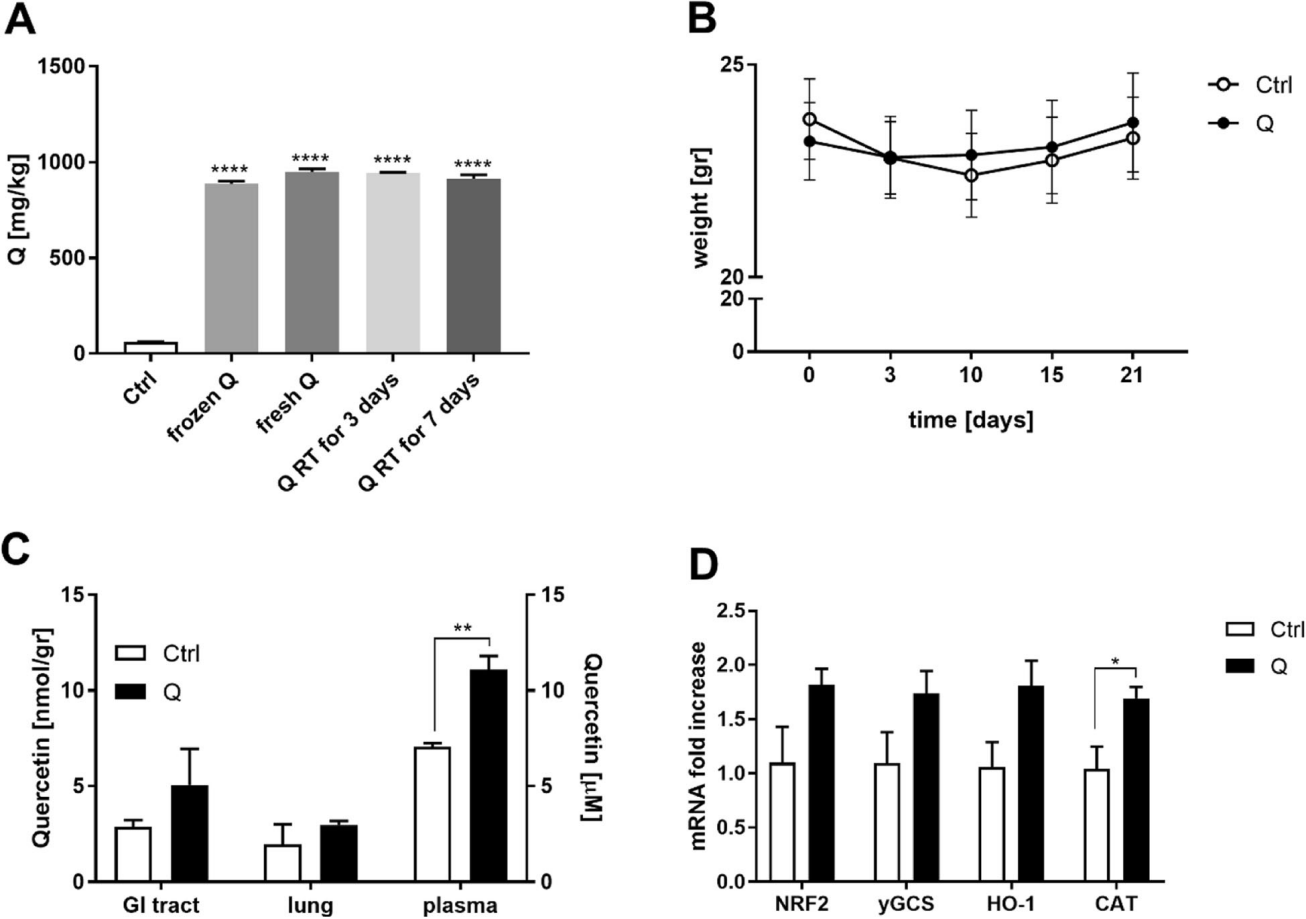
One-week dietary quercetin supplementation safely enhances plasma and pulmonary quercetin levels as well as pulmonary expression of Nrf2 and Nrf2-responsive genes. **a** quercetin concentration in differently stored diet aliquots, **b** weight gain in animals fed either the control (*n* = 3) or quercetin-enriched (*n* = 3) diet for 1 week, **c** quercetin concentrations in different organs after 1 week, **d** expression of Nrf2 and Nrf2-responsive genes after 1-week dietary intervention. Data are expressed as mean ± SEM; * = *P* < 0.05; ** = *P* < 0.01 (*n* = 3 per treatment)

Prior to applying the bleomycin challenge, the safety and effectiveness of the quercetin supplementation were assessed by analysing food intake as well as the body weight and overall survival of C57Bl/6 J mice from our in house breeding colony fed either the control or quercetin-enriched diet. As can be deducted from Fig. 2a, quercetin concentrations in the diet were stable throughout the study in both the frozen and room temperature pellets. Average body weight gain (Fig. 2b) was not affected by diet nor were food intake and survival (data not shown). This 1-week supplementation resulted in significantly higher plasma quercetin levels compared to those levels in animals fed the control diet (11.08 ± 0.73 μM versus 7.05 ± 0.2 μM; Fig. 2c, *p* < 0.01). However, quercetin supplementation did not significantly increase quercetin concentrations in the lungs (2.97 ± 0.21 nmol/g versus 1.95 ± 1.06 nmol/g) and GI tract (5.05 ± 1.9 nmol/g versus 2.89 ± 0.35 nmol/g). Additionally, dietary supplementation of quercetin for 1 week increased the pulmonary expression levels of Nrf2 and the Nrf2-responsive genes ƴ-GCS, HO-1 and CAT of which the latter was significantly enhanced by the quercetin diet (Fig. 2d, *p* < 0.05).

### Quercetin affects BLM-induced pulmonary fibrogenesis

**Fig. 3:**
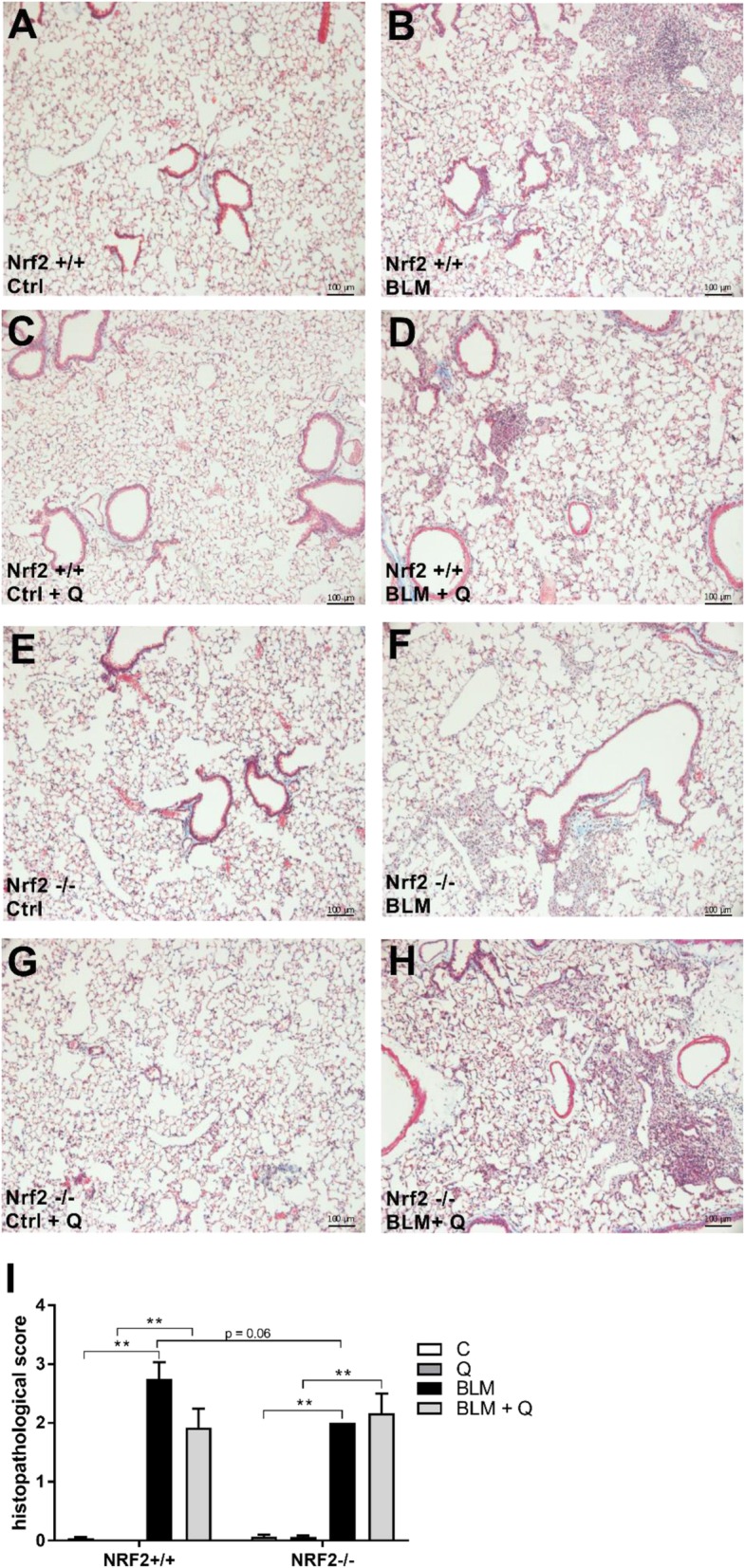
Single pharyngeal bleomycin instillation induces inflammatory lesions with some focal increase of collagenous fibers in Nrf2^−/−^ mice to a lesser extent than in Nrf2^+/+^ mice. Effects of bleomycin treatment in representative Nrf2^+/+^ and Nrf2^−/−^ mice are shown for (**a**-**h**) Masson Trichome staining of lung tissue showing nuclei (dark brown), cytoplasm (pink) and collagen (blue) and (**i**) histopathological score of the lung tissue (*n* = 5–6 mice/group). Data are expressed as typical example (panel A-H) or mean ± SEM (panel I); * *P* < 0.05; ** *P* < 0.01

Upon verifying the pulmonary and systemic uptake of quercetin when supplemented via the diet over a period of 1 week in an independent pilot study, Nrf2^−/−^ and Nrf2^+/+^ mice received a single bleomycin challenge via pharyngeal aspiration to induce pulmonary fibrosis. In this model, fibrosis has been found to develop from 7 to 10 days and peaks at 14–21 days after bleomycin injury [36]. Two weeks after the bleomycin challenge, histological examination of H&E as well as Masson’s Trichrome-stained sections of the lungs revealed a thickening of the airway epithelium in several bleomycin-treated Nrf2^+/+^ and Nrf2^−/−^ mice. However, a final assessment of these finding was not possible due to some variation during the pharyngeal instillation. Additionally, all lungs of bleomycin-treated Nrf2^+/+^ and Nrf2^−/−^ mice revealed multi-focal inflammatory lesions, predominantly in the more central region around the main airways (Fig. 3a-h). These inflammatory lesions mainly consisted of mononuclear cells, fibroblasts and alveolar macrophages that partly appeared as foam cells. Individual grading of these inflammatory lesions resulted in a significantly higher score in the bleomycin-treated Nrf2^+/+^ and Nrf2^−/−^ mice compared to their control littermates (Fig. 3i). In some areas of inflammation, a minimal increase of collagenous fibers was detected. Morphological discrimination between the histological changes occurring in bleomycin-treated wildtype animals compared to their knockout littermates was both qualitatively and quantitatively impossible. Interestingly, there was a tendency of less inflammatory lesions in the Nrf2^+/+^ animals treated with quercetin compared to the control group (2.8 ± 0.7 versus 1.9 ± 0.8) although due to the high variation in individual scoring this difference did not reach statistical significance (*p* = 0.06). In Nrf2^−/−^ animals, no effect of the dietary antioxidant on either histology (Fig. 3f versus Fig. 3h) or inflammatory lesions (Fig. 3i) was observed suggesting that potential ameliorating effects of quercetin on the lung require a functional Nrf2 response.

To our surprise, the bleomycin treatment failed to cause more pronounced multi-focal inflammatory lesions in the Nrf2^−/−^ mice compared to their wildtype littermates as was expected based on previous studies in which the pro-fibrotic effects of this drug were indeed augmented in animals lacking a functional copy of Nrf2 [18, 37]. As in our hands the histological changes due to bleomycin were not different in the Nrf2^−/−^ animals than in their wildtype littermates (Fig. 3), it did not deem relevant to further expand molecular analyses of the Nrf2^−/−^ in the present pilot study that was primarily designed to investigate the health beneficial effects of quercetin in a model of pulmonary fibrogenesis.

**Fig. 4:**
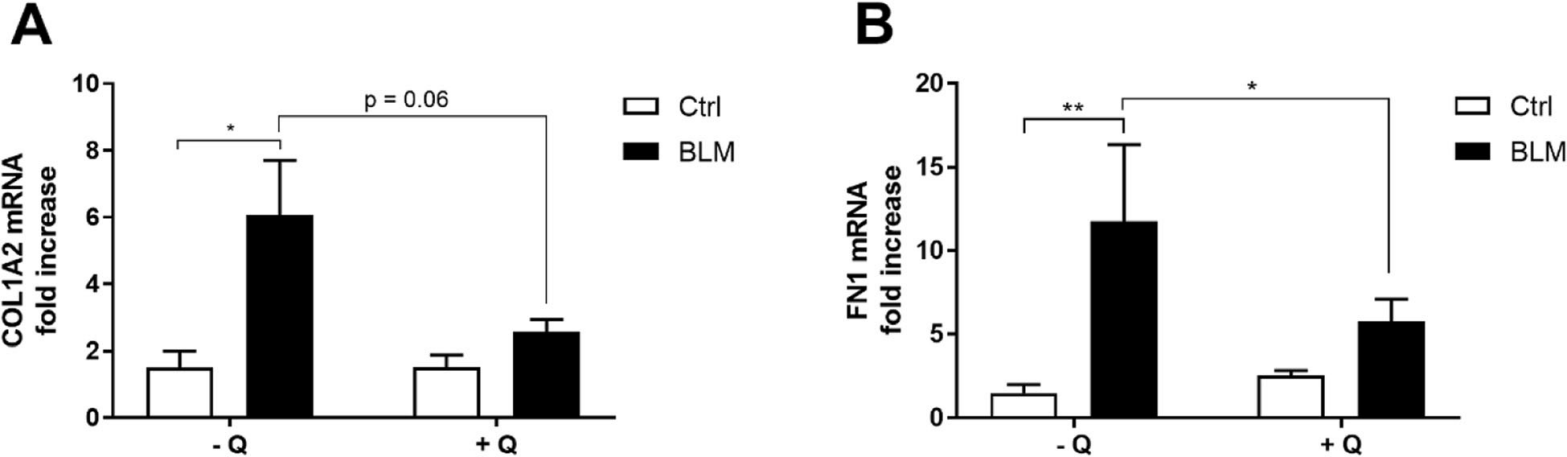
Quercetin-enriched diet reduces fibrogenic markers in Nrf2^+/+^ mice instilled with bleomycin. Effects of bleomycin treatment in the absence or presence of dietary quercetin supplementation are shown for pulmonary expression of (**a**) COL1A2 and (**b**) FN1. Data are expressed as mean ± SEM; *P* < 0.05; ** *P* < 0.01

In the Nrf2^+/+^ mice, the extracellular matrix (ECM)-related markers collagen (COL1A2) and FN1 supported the histopathology as their gene expression revealed a respectively 3-fold (Fig. 4a, *P* < 0.05) and 10-fold increase (Fig. 4b, *P* < 0.01) in bleomycin-treated animals compared to the control group. Interestingly, the expression levels of both COL1A2 and FN1 were lower in the bleomycin-treated Nrf2^+/+^ animals fed the quercetin diet compared to those fed the control diet (Figs. 4a, *P* = 0.06 and Fig. 4b, *P* < 0.05).

Once the protective effects of quercetin against bleomcyin-induced inflammatory lesions and fibrogenic hallmarks were established, their link with the anti-inflammatory and antioxidative capacities of this dietary antioxidant were evaluated.

### Quercetin reduces pulmonary yet not systemic BLM-induced inflammation

**Fig. 5:**
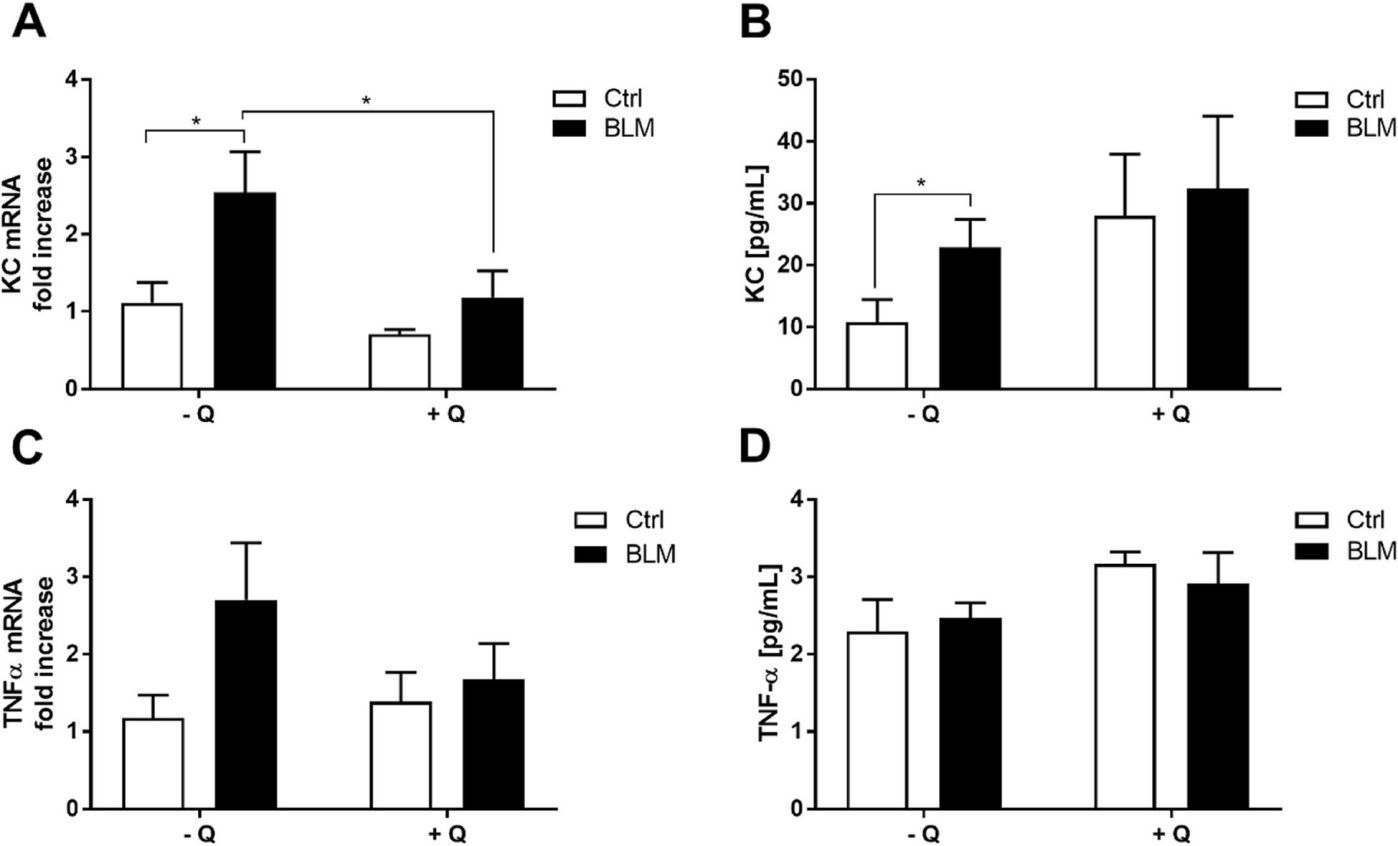
Anti-inflammatory effects of quercetin are associated with pulmonary but not systemic anti-inflammatory capacities. Pulmonary gene expression and systemic plasma levels of KC (**a** and **c**) and TNF-α (**b** and **d**) upon bleomycin challenge in the absence or presence of dietary quercetin supplementation. Data are expressed as mean ± SEM, * = *P* < 0.05

As depicted in Fig. 5, the pulmonary expression of the inflammatory genes KC (panel A) and TNF-α (panel C) was two times higher upon bleomycin treatment (*P* < 0.05 for KC; n.s. for TNF-α) and restored to baseline levels in the quercetin-fed group (*P* < 0.05 for KC; n.s. for TNF-α). Systemic plasma levels of these cytokines revealed that KC levels significantly increased from 10.81 ± 3.58 pg/ml to 22.88 ± 4.50 pg/ml upon bleomycin treatment (Fig. 5b) whereas TNF-α levels remained unaffected by this pro-fibrotic drug (Fig. 5d). Surprisingly, quercetin supplementation did not exert any systemic anti-inflammatory effects as both the plasma KC (Fig. 5b) and TNF-α (Fig. 5d) levels were unaltered by the antioxidant without and with bleomycin challenge. The other cytokines measured in the plasma were under the detection limit.

### Quercetin decreases levels of BLM-induced oxidative parameters

**Fig. 6:**
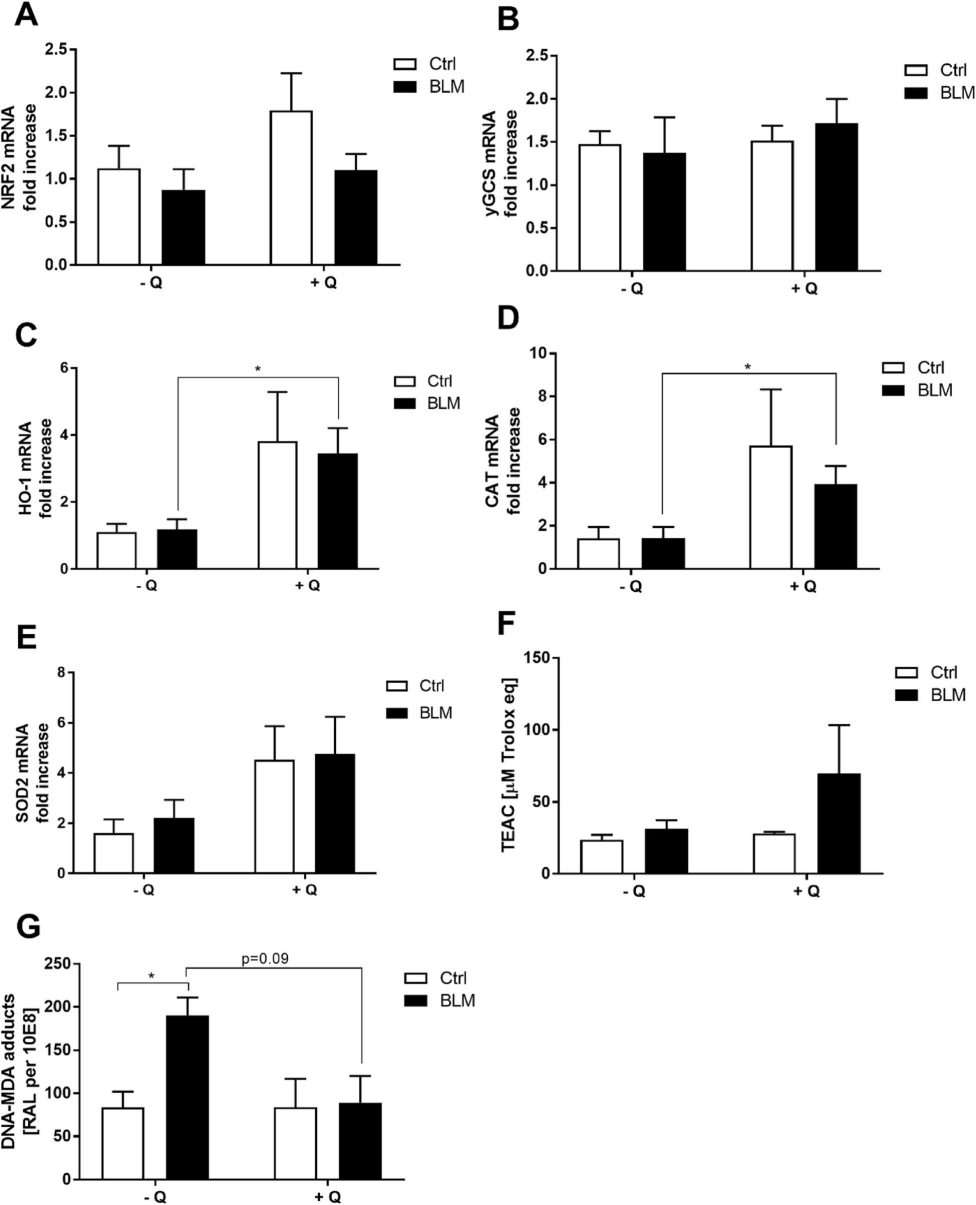
Anti-fibrotic effects of quercetin are associated with its anti-oxidative capacities. Pulmonary gene expression levels of (**a**) Nrf2, (**b**) γGCS, (**c**) HO-1, (**d**) CAT, (**e**) SOD2, (**f**) plasma total antioxidant status and (**g**) DNA-MDA adducts upon bleomycin challenge in the absence or presence of dietary quercetin supplementation. Data are expresses as mean ± SEM; * = *P* < 0.05

Since quercetin is a pronounced dietary antioxidant, its influence on both the enzymatic and non-enzymatic antioxidants was evaluated as well as the possible protection it could offer against oxidative DNA damage. Pulmonary expression of the Nrf2-responsive gene ƴ-GCS was not affected by either the pro-fibrotic drug or the dietary antioxidant whereas quercetin supplementation did significantly increase the pulmonary expression of Nrf2 (1.80 ± 0.43) as well as the Nrf2-responsive genes HO-1 (3.82 ± 1.46), catalase (5.73 ± 2.60) and SOD2 (4.52 ± 1.35) (Fig. 6a-e; *P* < 0.05). Interestingly, bleomycin did not affect the pulmonary expression levels of Nrf2, ƴ-GCS, catalase or SOD2 as reflected in baseline levels in the lungs of animals fed the control diet and enhanced levels again in the animals receiving the quercetin-supplemented diet (Fig. 6a-d, *P* < 0.05 for all genes except SOD2).

The systemic plasma antioxidant defences were evaluated by means of the TEAC assay that analyses the total antioxidant status. Neither quercetin alone nor bleomycin had an effect on the systemic antioxidant status, but once combined they tended to enhance the total plasma antioxidant capacity (28.07 ± 5.84 vs 69.78 ± 33.66 μM) but this effect was very variable between individual mice (Fig. 6f). To assess whether quercetin cannot only boost the antioxidant defences but also offer protection against oxidative damage, DNA-MDA adduct levels were analysed. As anticipated, the pro-fibrotic trigger bleomcyin significantly increased DNA-MDA adduct levels (83.8 ± 18.26 versus 190.2 ± 20.65 RAL per 10^8^) (Fig. 6g). Dietary supplementation of the antioxidant quercetin restored these elevated DNA-MDA levels back to baseline levels (89.34 ± 30.78 RAL per 10^8^) although this effect did not reach statistical significance (Fig. 6g, *p* = 0.09).

## Discussion

IPF is associated with the presence of oxidative stress in the lungs and IPF patients display various markers of oxidative damage [38, 39] and a downregulation of several antioxidants [40–42]. Restoration of this imbalance between oxidants and antioxidants has been suggested as potential treatment strategy for IPF [43].

In the present study, we provide evidence that dietary quercetin supplementation protects against bleomycin-induced fibrogenesis in mice. The results from our kinetic investigations showed that dietary supplementation of quercetin significantly increased the plasma quercetin concentration and enhanced pulmonary antioxidant gene expression without affecting the weight and survival of the mice. It was anticipated that Nrf2 has no influence on the actual uptake of this dietary antioxidant or the pulmonary quercetin levels achieved. However, further investigations are needed to completely rule this out. In the Nrf2 pilot study, bleomycin instillation induced changes in the lung structure, associated with multi-focal inflammatory lesions and focal collagen deposition as well as profibrotic gene expression. This bleomycin-induced pulmonary damage could partly be rescued by dietary quercetin supplementation. Furthermore, quercetin supplementation induced upregulation of Nrf2 and Nrf2-regulated genes and slightly reduced oxidative DNA damage in the bleomycin-challenged lungs. However, histopathologically no clear treatment associated differences could be detected.

Although quercetin has previously been reported to exert anti-inflammatory effects via modulation of Nrf2-signaling, these observations have mainly been made in models of acute lung injury or viral and bacterial infections [44] [45, 46]. Only a few studies have investigated the Nrf2-modulating effect of quercetin in relation to fibrosis, but these studies have focused on other organs including the heart [47] and liver [48]. Thus far, only one study has been published investigating the influence of quercetin on pulmonary Nrf2-activation, showing that, in line with our findings, in vitro quercetin could reduce collagen deposition in healthy lung fibroblasts via increased Nrf2 translocation and enhanced HO-1 expression [49]. To the best of our knowledge, our study is the first to demonstrate quercetin exerts anti-fibrogenic and anti-inflammatory effects in vivo in pulmonary fibrosis and that these effects are partly due to Nrf2-modulation.

Antioxidant food supplements such as quercetin have been shown to reduce markers of oxidative stress and fibrotic gene expression in vitro [28, 49]. At present, this is the first study that addressed bleomycin-induced fibrogenesis in mice after ad libitum dietary supplementation of quercetin. Until now, there have only been two studies investigating the effects of solely quercetin supplementation in the absence of any regular drugs or other supplements. However, these studies differed from our study with respect to the animal model applied and the administration mode of quercetin. In agreement with our results, quercetin demonstrated positive effects in two other animal models of pulmonary fibrosis [50, 51]. Oral quercetin supplementation (100 mg/kg bodyweight per day) significantly reduced the hydroxyproline content in the lungs of young rats (10–12 weeks) instilled with bleomycin. This antifibrotic effect was accompanied by decreased levels of MDA, a marker for lipid peroxidation, and enhanced catalase and superoxide dismutase activity [50]. In aged mice (> 12 months), intraperitoneally quercetin treatment (30 mg/kg every other day) 7 days after bleomycin instillation reduced hydroxyproline content in the lungs as well. In this model, the antifibrotic effect of quercetin was combined with decreased pulmonary markers of senescence which in turn reduced the apoptosis resistance of pulmonary fibroblasts [51], indicating that quercetin can also reverse the apoptosis-resistant phenotype of fibroblasts in IPF.

An important hallmark of fibrosis is inflammation and it has been shown before that quercetin has anti-inflammatory capacities and for instance is able to decrease inflammatory cell infiltration such as macrophages, neutrophils and eosinophils [50]. Our findings suggest that quercetin exerts an anti-inflammatory effect partly via reducing the pulmonary expression of the inflammatory cytokine KC of which the human functional homologue CXCL1 is known to be upregulated in BALF from IPF patients during acute exacerbations [52]. Other studies have suggested that bleomycin also increases TNFα [50, 53], which was not observed in our study.

Alleged mode of action underlying this observed defence against bleomcyin-induced pulmonary damage is the antioxidative capacity of quercetin as it is known that bleomycin evokes excessive ROS formation [36, 54] and that the lungs are especially vulnerable for alterations in the redox balance [13, 36]. Indeed, already in the absence of bleomycin dietary quercetin supplementation led to enhanced pulmonary expression of the redox-sensitive transcription factor Nrf2, as well as Nrf2-responsive antioxidant genes including catalase, HO-1 and ƴ-GCS, previously shown to protect against bleomycin-induced pulmonary fibrosis [18, 22, 55]. In the presence of bleomycin, quercetin supplementation increased the gene expression of most Nrf2-responsive endogenous antioxidants but due to high variation this effect was only statistically significant for catalase and HO-1. This increased antioxidant gene expression was associated with a slightly enhanced total plasma antioxidant capacity in bleomycin-exposed animals fed the quercetin diet compared to animals triggered with only bleomycin, although statistical significance was again not reached due to rather high variation. In line with these increased antioxidative defences due to dietary quercetin supplementation, the DNA-MDA adduct levels were restored to baseline levels in the bleomycin-treated animals fed the quercetin diet compared to their exposed littermates receiving the control diet. Previous studies have revealed that these adduct levels are an important biomarker of oxidative damage with premutagenic activity [56, 57], suggesting that lowering these levels might be associated with beneficial health effects.

Surprisingly, histological examination of the bleomycin-challenged lungs of Nrf2^−/−^ mice alongside their Nrf2^+/+^ wildtype littermates tended to reveal less inflammatory lesions in the Nrf2 deficient animals. This seems in contrast with previous studies that reported aggravated bleomycin-induced fibrogenesis in animals lacking this transcription factor [18, 37]. Various reasons can be provided for these unexpected differences between bleomycin-induced fibrogenesis in Nrf2^−/−^ mice in our study versus previously published studies [22, 37] including differences in bleomycin dose, time point of analysis and intrinsic differences in mouse models [36]. For instance, the background of the animals used in the present study is C57BL6 whereas Cho et al have studied the influence of Nrf2 on bleomycin-induced fibrogenesis in ICR/Sv129-Nrf2 mice [22]. Interestingly, slower but more persistent destructive fibrotic responses to bleomcycin have been reported in Sv129 mice compared to C57BL6 mice [58]. It is beyond the scope of the present study to further investigate the apparently contradictive findings with respect to the effects of bleomycin in Nrf2 deficient mice strains, but other studies have also already indicated that Nrf2 can exert anti-protective effects under specific conditions. Interestingly, in this regard Nrf2 has been found to play a pivotal role in the activation of the NOD-like receptor pyrin domain-containing-3 (NLRP3) inflammasome, which was demonstrated to be crucial for bleomycin-induced fibrogenesis [59, 60]. Taking these findings into consideration, knocking down Nrf2 would imply less NLRP3-induced inflammation and fibrogenesis upon bleomycin-induced ROS formation, and thus align with the observations in our present study.

Nevertheless, our current observation that the histopathology of bleomycin-challenged lungs of Nrf2^−/−^ mice with and without quercetin diet did not differ significantly, supports the concept that quercetin inhibits fibrogenesis at least partly by boosting the Nrf2 antioxidant pathway [28].

In contrast to our findings, a recent study demonstrated no upregulation of Nrf2 genes after quercetin supplementation [51], however, this study has been performed in aged mice indicating that quercetin alone cannot upregulate Nrf2 in this model. It has been indicated before that bronchial epithelial cells from elderly individuals express increased levels of negative Nrf2 regulators [61] suggesting that only targeting the redox balance via inducing Nrf2 is not enough to counteract the development of fibrosis in IPF patients. Therefore, the possible protection offered by quercetin has to be elucidated in a different animal model of pulmonary fibrosis.

Our findings indicate that quercetin is well tolerated and taken up but only minimally reduced markers of fibrosis. Possible explanations for the observed effects of the dietary supplementation being less pronounced than anticipated include i) the dose and mode of quercetin administration, or ii) the way of modulating the redox balance. It could be possible that the concentration in the lungs was not high enough due to the fact that quercetin is metabolized in the liver upon absorption by the small intestine. The average intake of quercetin in our study was approximately 200 mg quercetin/kilogram BW and resulted in a quercetin concentration of 11.08 ± 0.73 μM in the plasma, 2.97 ± 0.21 nmol/g in the lungs and 5.05 ± 1.9 nmol/g in the GI tract which was comparable to a different study performed in rats [33]. In this study of de Boer et al, the diet was supplemented with 1% quercetin for 11 weeks, which equals an approximate intake of 500–800 mg/kg bodyweight [33]. The highest concentration of quercetin was found in the plasma and lungs in (40.5 μmol/L and 5.02 nmol/g tissue respectively) which is comparable to the findings in our study taking the differences in administered quercetin concentrations into account. However, it is important to note that in both studies the quercetin levels were measured after ex vivo enzymatic hydrolysis whereas the antioxidant is primarily present conjugated to methoxy, sulfate and glucoronic acid in tissues. It can yet not be excluded that quercetin is also present in its unconjugated form as especially in the lung relatively high levels of the aglycone were measured, presumably due to high pulmonary deconjugation activity [33]. Although most quercetin metabolites possess some antioxidative capacities themselves [17, 62], the aglycone is the strongest antioxidative and thus most desirable form of quercetin to expect health beneficial effects from. Consequently, it would be of interest to investigate alternative ways of quercetin administration to prevent the uptake in the GI tract and improve efficiency. Therefore, direct pulmonary administration of quercetin to increase its uptake in the target organ without being metabolized may be more beneficial compared to orally delivered quercetin to exert stronger beneficial effects than currently observed. Indeed, quercetin intratracheally applied reduced LPS-evoked lung inflammation in mice via an HO-1 dependent pathway [63]. Interestingly, other more direct administration routes have also been reported to lead to successful quercetin interventions as for instance intraperitoneal administration was shown to suppress bleomycin-induced lung injury and inflammation in hamsters [64]. Additionally, other studies have reported that quercetin, either administered orally via gavage [65] or intravenously as liposome [66], exerts anti-inflammatory effects in bleomycin-induced pulmonary fibrosis in mice. An important factor involved in these stronger beneficial effects is the direct and sometimes even “bolus” application of quercetin which not only results in different toxicokinetics compared to dietary administration but is also more difficult to translate to human conditions. Moreover, part of the beneficial effects of our oral administration being only moderate could be explained by a possible variation in the daily quercetin intake per individual mouse which could also explain the variances observed within the quercetin group.

Second possible explanation for the moderate effects of the dietary modulation observed in the present study is that the quercetin intervention was too subtle and specific to exert pronounced effects on the pro-fibrotic and especially pro-inflammatory outcomes of the bleomycin challenge. Quercetin is a potent scavenger of hydroxyl radicals and superoxide [67, 68] and to a lesser extent of H_2_O_2_ [17]. Since NADPH oxidase (NOX) 4, one of the important pulmonary ROS sources that is upregulated in IPF and promotes alveolar epithelial cell death [69] and fibroblast activation [70, 71], mainly produces H_2_O_2_ it can be suggested that quercetin cannot counteract NOX4-induced fibrotic outcomes.

Instead of scavenging excessive production of specific ROS by adding one particular antioxidant to the diet, modulating the redox balance in a more delicate way including a combination of components or a variety of redox-sensitive targets might be more effective to exert beneficial effects in a disease associated with oxidative stress. Such a possible redox-sensitive target is Nrf2, already proven to protect against bleomycin-induced pulmonary fibrosis, as it has recently been shown that this transcription factor reduces epithelial mesenchymal transition which is a key progression that promotes pulmonary fibrosis [55]. Interestingly, the currently for IPF therapy approved drug perfinidone that affects intracellular antioxidants, inflammatory cytokines secretion and collagen synthesis exerts its antioxidative capacities via Nrf2 activation [54]. This is in line with the concept previously postulated that Nrf2 may offer greater protection as therapeutic target in future IPF treatment than an antioxidant strategy involving a single antioxidant that has not been effective thus far [72]. As quercetin is both a ROS scavenger and a potential activator of Nrf2 and Nrf2-responsive genes [72, 73], combining regular pirfenidone or nintendanib treatment with this dietary compound may effectively modulate redox balance in IPF and optimize treatment outcome in these patients. Of additional interest to the effectiveness of this combination would be the fact that quercetin and its metabolites exert their effects also via modulating the activity of protein kinases including phosphoinositide 3-kinase [74], Akt/protein kinase B [75] as well as SRC family kinase members SRC, LYN and FYN [76]. Intriguingly, a recent study described that quercetin in combination with the SRC/ABL protein kinase inhibitor dasatinib reverses bleomycin-induced pulmonary fibrosis in aged mice through the reduction of various senescence markers, thereby improving lung function [77]. These results further underline that quercetin might exert its maximal health beneficial effects in combination with other anti-fibrotic drugs, indicating that combination therapy might be more beneficial to counteract ROS-induced damage and the underlying inflammation.

Among the limitations of the present study are the relatively low number of animals per group, the exclusive use of the left lung for histology, the variable pharyngeal bleomycin instillation leading to a rather large spread in the data, the animal model applied and the single quercetin concentration tested. Indeed, the possible protection offered by quercetin remains to be elucidated in a different animal model of pulmonary fibrosis applying larger groups as well as a different trigger. Although none of the existing murine models of pulmonary fibrosis really reflects the complex pathogenicity of this disease in humans, specific criticism on the bleomycin model includes the pronounced and rather prolonged inflammatory response whereas this process is thought to only play a minor role in IPF development [36, 78]. However, it is thought that inflammation may play a role, though potentially to a different extent, in both murine and human pulmonary fibrosis, indicating that more research is needed to improve understanding the involvement of inflammation in IPF [78]. Additionally, different time points and doses for bleomycin administration have been reported in literature, resulting in different degrees of fibrotic damage and inflammation and hampering the straight-forward comparison between various studies [36]. Finally, the current study only tested one quercetin dose, i.e. 800 mg quercetin per kg diet, whereas the possible healthy beneficial effects of this dietary antioxidant may of course be dose-dependent. However, the current set-up was a proof-of principle pilot study to evaluate the possible biological effects of a dietary compound combining the toxicological principle that no lower doses have to be tested if the highest dose is not biologically effective with the 3R policy in conducting animal studies. Interestingly, the applied dose was selected based on previous work by de Boer et al [33] that showed a dose-dependent increase in pulmonary quercetin levels when different doses were given, suggesting it would be very interesting to test additional lower doses in a future study now that our pilot study has observed positive effects for a very high dose.

## Conclusion

In conclusion, we demonstrated that dietary quercetin supplementation reduces pro-fibrotic gene expression after bleomycin instillation in lungs of mice. Additionally, a tendency of decreased inflammatory lesions was seen. However, the observed protective effects were only moderate indicating that nutritional ROS scavenging strategies are not enough to counteract the development of pulmonary fibrosis.

## Data Availability

The datasets used and/or analyzed during the current study are available from the corresponding author on reasonable request.

## Abbreviations

ARE: Antioxidant responsive element
BALF: Broncho-alveolar lavage fluid
BLM: Bleomycin
BW: bodyweight
CAT: Catalase
COL1A2: Collagen1a2
FDA: Food and drug administration
FN1: Fibronectin 1
H&E: Hematoxylin and eosin
HO-1: Heme oxygenase 1
IPF: Idiopathic pulmonary fibrosis
IL: Interleukin
KC: Keratinocyte chemoattractant
MDA: Malondialdehyde
MIP: Macrophage inflammatory protein
NAC: N-acetyl cystein
NOX: NADPH oxidase
Nrf2: Nuclear factor (erythroid-derived 2)-like 2
ROS: Reactive oxygen species
SOD2: Superoxide dismutase 2
TNF-α: Tumor necrosis factor alpha
Ƴ-GCS: Gamma glutamyl cysteine synthase
